# Increasing the frequency of hand washing by healthcare workers does not lead to commensurate reductions in staphylococcal infection in a hospital ward

**DOI:** 10.1186/1471-2334-8-114

**Published:** 2008-09-02

**Authors:** Clive B Beggs, Simon J Shepherd, Kevin G Kerr

**Affiliations:** 1 School of Engineering, Design and Technology, University of Bradford, Bradford, BD7 1DP, UK; 2 Department of Microbiology, Harrogate and District NHS Foundation Trust, Harrogate District Hospital, Lancaster Park Road, Harrogate, HG2 7SX, UK

## Abstract

**Background:**

Hand hygiene is generally considered to be the most important measure that can be applied to prevent the spread of healthcare-associated infection (HAI). Continuous emphasis on this intervention has lead to the widespread opinion that HAI rates can be greatly reduced by increased hand hygiene compliance alone. However, this assumes that the effectiveness of hand hygiene is not constrained by other factors and that improved compliance in excess of a given level, in itself, will result in a commensurate reduction in the incidence of HAI. However, several researchers have found the law of diminishing returns to apply to hand hygiene, with the greatest benefits occurring in the first 20% or so of compliance, and others have demonstrated that poor cohorting of nursing staff profoundly influences the effectiveness of hand hygiene measures. Collectively, these findings raise intriguing questions about the extent to which increasing compliance alone can further reduce rates of HAI.

**Methods:**

In order to investigate these issues further, we constructed a deterministic Ross-Macdonald model and applied it to a hypothetical general medical ward. In this model the transmission of staphylococcal infection was assumed to occur after contact with the transiently colonized hands of HCWs, who, in turn, acquire contamination only by touching colonized patients. The aim of the study was to evaluate the impact of imperfect hand cleansing on the transmission of staphylococcal infection and to identify, whether there is a limit, above which further hand hygiene compliance is unlikely to be of benefit.

**Results:**

The model demonstrated that if transmission is solely via the hands of HCWs, it should, under most circumstances, be possible to prevent outbreaks of staphylococcal infection from occurring at a hand cleansing frequencies < 50%, even with imperfect hand hygiene. The analysis also indicated that the relationship between hand cleansing efficacy and frequency is not linear – as efficacy decreases, so the hand cleansing frequency required to ensure R0 < 1 increases disproportionately.

**Conclusion:**

Although our study confirmed hand hygiene to be an effective control measure, it demonstrated that the law of diminishing returns applies, with the greatest benefit derived from the first 20% or so of compliance. Indeed, our analysis suggests that there is little benefit to be accrued from very high levels of hand cleansing and that in most situations compliance > 40% should be enough to prevent outbreaks of staphylococcal infection occurring, if transmission is solely via the hands of HCWs. Furthermore we identified a non-linear relationship between hand cleansing efficacy and frequency, suggesting that it is important to maximise the efficacy of the hand cleansing process.

## Background

Hand hygiene is generally considered to be the most important measure that can be applied to prevent the spread of healthcare-associated infection (HAI) [1]. Through regular cleansing of hands, healthcare workers (HCWs) reduce the risk to transmitting nosocomial pathogens between patients and thus reduce the risk of exogenously-acquired infection. This has lead to the widespread opinion that HAI rates can be greatly reduced by increased hand hygiene compliance alone [2], with the result that healthcare authorities around the world have vigorously promoted hand hygiene as the pre-eminent measure in controlling HAI. However, this approach assumes that the effectiveness of hand hygiene is not limited by other factors and that greater compliance will continue to yield improved results. While it is undoubtedly the case that improved hand hygiene is beneficial [1,3], there is growing evidence that increased compliance may not yield the hoped for results. For example, using dynamic transmission models, Cooper *et al *[4] and McBryde *et al *[5] found that the law of diminishing returns applies to hand hygiene, with the greatest benefits occurring in the first 20% or so of compliance. Furthermore, Austin *et al *[6] and Beggs *et al *[7] demonstrated that poor cohorting of nursing staff profoundly influences the effectiveness of hand hygiene measures. Moreover, Grundmann *et al *[8] found that during periods of under-staffing, it is necessary to greatly increase the frequency of hand cleansing in order to prevent outbreaks of infection. Collectively, these findings raise intriguing questions about the limitations of hand hygiene and the extent to which increasing compliance alone can further reduce the spread of infection. In order to investigate this issue further, we constructed a deterministic Ross-Macdonald model to analyse the hypothetical general medical ward presented by Cooper *et al *[4] who found that hand hygiene compliance levels > 30% made little impact on the prevalence of *Staphylococcus aureus *infection on the ward. However, in their study they assumed that each hand cleansing event had an efficacy of 100%, – which in "real life" situations on a busy hospital ward is unlikely to be the case. Given this, we decided to repeat the study of Cooper *et al *by using a model that takes into account not only the frequency of handwashing but also the efficacy of the hand cleansing process. The aim of the study was to evaluate the impact of imperfect hand cleansing on the transmission of staphylococcal infection and to identify whether there is a limit, above which further compliance would be unlikely to yield beneficial results.

Since Ignaz Semmelweis reported in 1847 that the incidence of puerperal fever in an obstetric unit could be drastically reduced through handwashing [9], cleansing of hands has been the principal measure employed in hospitals to reduce HAI. Until recently, hand cleansing was achieved through the use of soap and water, a time consuming [10] and sometimes inefficient process. However, in recent years, new alcohol-based products have become widely available. These are more convenient and quicker to use than soap and water and their use is being vigorously promoted. Despite this, levels of hand hygiene compliance remain low; typically < 50% [11-13]. One reason for low compliance appears to be the large number of handwashing opportunities that arise during patient care. These make it difficult for HCWs to cleanse their hands effectively, while still carrying out their clinical duties. For example, Pittet *et al *[10] observed an average of 43.4 hand hygiene opportunities per hour of patient care on an intensive care unit (ICU), which suggests that busy staff have very little spare time in which to cleanse their hands.

Although there are relatively few data on the types of patient-care activities that result in transmission of pathogens on the hands of HCWs, there is clear evidence that such transmission does occur. In a study of 'clean' activities, such as lifting patients, palpation of pulses or during sphygmomanometry, Casewell and Phillips [14] found that nurses could contaminate their hands with 100–1000 colony forming units (cfu) of *Klebsiella *spp. Similarly, Ehrenkranz and Alfonso [15] found that nurses readily contaminated their hands (i.e. 10–600 cfu/mL in glove juice samples) by touching the groins of patients heavily colonised with *Proteus mirabilis*. This suggests that contamination of the hands of HCWs is a frequent occurrence, and that relatively innocuous procedures can result in transient colonization. This is supported by the results of a study undertaken before the use of gloves by HCWs became common practice, which reported that 15% of nurses working in an isolation unit carried a median of 10^4 ^cfu of *S. aureus *on their hands and 29% of nurses working in a general hospital had a median count of 3.8 × 10^3 ^cfu [16]. In another study, Daschner [17] found that *S. aureus *could be recovered from the hands of 21% of HCWs on an ICU, and that 21% of doctors and 5% of nurse carriers had > 1,000 cfu of the organism on their hands. Collectively, these data reveal that the hands of HCWs can become heavily contaminated when undertaking clinical procedures and they reinforce the need to maintain good hand hygiene procedures.

Hand cleansing is an imperfect process, the efficacy of which depends on the product used, the technique employed, and the duration of the process. Thus it is likely that it will not remove all the microorganisms from the hands of HCWs. Girou *et al *[18], for example, found that hand rubbing with a 75% alcohol-based solution resulted in a median percentage reduction in bacterial contamination of 83% compared with a reduction of only 58% when washing with medicated soap. Similarly, Zaragoza *et al *[19] found that the use of an alcoholic solution resulted in an average reduction in cfu of 88.2% compared with only 49.6% when soap and water was used.

## Method

In order to investigate the impact of sub-optimal hand cleansing on the transmission of staphylococcal infection, we modified the model of Cooper *et al *[4], so that it considered both handwashing frequency and handwashing efficacy (see Appendix) allowing us to simulate the effect of imperfect hand cleansing, something which other investigators had overlooked in their respective studies [3-6,20].

In order to permit direct comparison with Cooper *et al *[4], we used the same data and simulated the same general medical ward as they did. Definitions of the variables used in our model are presented in Table 1.

**Table 1:** Parameters and their default values

Parameter	Meaning	Default value
N	Number of patients	20
n'	Number of health care workers (HCWs)	3
μ	Patient removal rate	0.10 per day
μ'	Handwashing rate	14.0 per day
λ'	Average efficacy of each handwashing event	0.5 (i.e. 50%)
γ	Detection rate of colonized patients	0.10 per day
σ	Proportion of admissions already colonized	0.01
c	Patient-HCW contact rate	5 per patient per HCW per day
p	HCW-patient transmission probability (i.e. transmissibility)	0.1
p'	Patient-HCW transmission probability (i.e. transmissibility)	0.1
β	HCW-patient transmission rate (β = cp)	0.5
β'	Patient-HCW transmission rate (β' = cp')	0.5

In the model the rate of change of colonized patients, *y*, on the ward is given by:

(1) $$\frac{dy}{dt}=\sigma \left(\mu x+\mu y+\gamma y\right)+\beta x\frac{y^{\prime }}{n^{\prime }}-y(\mu +\gamma )$$

and the rate of change of contaminated HCWs, *y'*, is:

(2) $$\frac{dy^{\prime }}{dt}=\beta^{\prime }y\frac{x^{\prime }}{n^{\prime }}-\mu^{\prime }\lambda^{\prime }y^{\prime }$$

In the model these differential equations are used to simulate the spread of staphylococcal colonization on the ward, with the basic reproductive number, *R*_0_, calculated using the following expression:

(3) $$R_{0}=\frac{(n-1)\beta \beta^{\prime }}{(\mu +\gamma )n^{\prime }\mu^{\prime }\lambda^{\prime }}$$

The basic reproductive number, *R*_0_, is the average number of secondary cases of colonization (which precedes infection) generated by one primary case in the absence of any infection control procedures.

Highly transmissible infections exhibit a large *R*_0_, whereas those which are less transmissible have a smaller value of *R*_0_. If the value of *R*_0 _is greater than 1, then each colonized patient will generate further new cases and it is likely that an outbreak will ensue. The outbreak will continue until *R*_0 _becomes less than 1, at which point it should begin to die out.

In the model the handwashing frequency, *f*_*h*_, is determined using the following expression derived by Cooper *et al *[4]:

(4) $$f_{h}=\frac{\mu^{\prime }}{\left(\mu^{\prime }+c\frac{n}{n^{\prime }}\right)}$$

In the model it was assumed that:

• The transmission of staphylococci is caused by contact with the transiently colonized hands of HCWs. Contacts between transiently colonized HCWs and uncolonized patients have a given probability of colonizing the patient, which is termed the HCW-to-patient transmissibility. In the model we did not consider the possibility of direct patient-to-patient contacts.

• HCWs acquire transient hand-contamination only by touching colonized patients. All such contacts between uncolonized HCWs and colonized patients have a given probability of colonizing the carer, which is termed the patient-to-HCW transmissibility. In the model we did not take into account direct HCW-to-HCW transmission or the possibility of HCWs becoming colonized from external sources.

• The HCW-to-patient transmissibility is assumed to be equal to the patient-to-HCW transmissibility.

• The population of patients is assumed to be homogeneous, with each patient considered equally likely to be in contact with a HCW in any time interval, equally likely to become colonized, and when colonized, equally likely to transmit the pathogen to a HCW on contact.

• The population of HCWs is assumed to be homogeneous. Variations between HCWs due to differences in behaviour (such as handwashing) and skin microflora are not considered.

• The detection of colonized patients is assumed to be a random process, with the mean detection time depending only on a constant level of surveillance activity.

• Once detected, colonized patients are assumed to be removed from the main ward and thus no longer a source of infection. Colonized patients who are not detected are removed from the ward at the same rate as uncolonized patients.

• Each time a HCW washes his or her hands only a portion of the transient microflora present is removed. The amount removed depends on the efficacy of the hand cleansing process.

• The efficacy of the hand cleansing process is assumed to be the same for all the HCWs on the ward.

In keeping with Cooper *et al *[4], we assumed the probability of a HCW contaminating their hands, or a patient becoming colonized, after each HCW-patient contact (i.e. the transmissibility value) to be 0.1. At the start of each simulation we assumed that all the patients on the ward were uncolonized and all HCWs were uncontaminated. Therefore, transmission could only begin once an infected/colonized patient had been admitted to the ward. The various events simulated in the model, together with their rates, are summarized in Table 2.

**Table 2:** Events and their rates

Event	Rate of event
Patient removal (when no colonization detected)	μ(x + y)
Detection of colonized patient and removal	γy
HCW hand cleaning	μ' (x' + y')
Removal of contamination from hands of HCWs	μ' y'λ'
HCW-patient contact	c(x + y)
HCW-to-patient transmission	βxy'/n'
Patient-to-HCW transmission	β' yx'/n'
Admission of uncolonized patient	(1 - σ) (μx + μy + γy)
Admission of colonized patient	σ(μx + μy + γy)

Where *x *represents the number of uncolonized patients, *y *represents the number of colonized patients, *y*' represents the number of HCWs with contaminated hands, and *x*' represents the number of HCWs whose hands are uncontaminated.

### Model Scenarios

In our study we modelled the effect of varying the handwashing frequency on the transmission of staphylococcal infection within the ward. For each frequency we modelled three possible efficacy scenarios (i.e. that each handwashing event removed 58%, 83% and 100% respectively of contaminants from the hands of HCWs). Efficacies of 58% and 83% were selected because they represent the values found by Girou *et al *[18] for HCWs using antibacterial soap and an alcohol-based solution, respectively, in a clinical setting. Having simulated the impact of hand cleansing frequency on the prevalence of infection, we then modelled the impact on *R*_0 _of changes in hand hygiene efficacy, contact transmissibility, and the HCW-patient contact rate.

## Results

**Figure 1:**
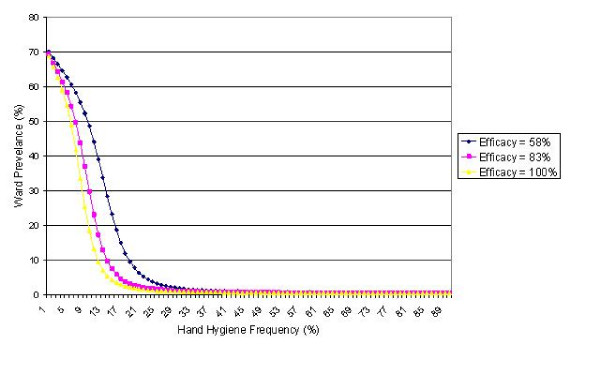
The impact of hand cleansing efficacy on cases of staphylococcal infection/colonization, where λ' = 58%, 83% and 100% respectively.

The impact of changes in hand cleansing efficacy on ward prevalence are presented in Figure 1. This shows the effect of variations in hand hygiene frequency for mean efficacy values of 58%, 83% or 100% respectively. It can be seen that as efficacy increases, so the frequency required to prevent an outbreak reduces. Indeed, under the default conditions stated in Table 1, very little benefit is accrued by increasing the hand cleansing frequency beyond 35%, even when soap and water is used to cleanse hands.

**Figure 2:**
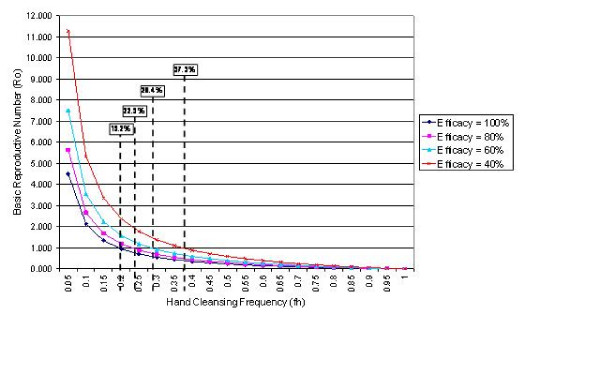
The relationship between the basic reproductive number, *R*_0_, and hand cleansing frequency, *f*_*h*_, for a range of hand cleansing efficacies (i.e. λ' = 100%, 80%, 60% and 40%). The broken lines represent to hand cleansing frequencies which equate to *R*_0 _= 1. Transmissibility is assumed to be 0.1.

Using equation 3 it is possible to examine the impact that variations in hand cleansing efficacy have on basic reproductive number, *R*_0_. The results of this analysis are presented in Figure 2, which shows *R*_0 _curves for four representative efficacy values (i.e. λ' = 100%, 80%, 60% and 40%). These show that even with very imperfect hand hygiene (i.e. λ' = 40%), it should be possible to prevent an outbreak of staphylococcal infection occurring at a hand cleansing frequency of, say 40%. Interestingly, Figure 2 suggests that the relationship between hand cleansing efficacy and frequency is not linear; rather, as the efficacy decreases, so the hand cleansing frequency required to ensure *R*_0 _< 1 increases disproportionately. This suggests that it is important to maximise the efficacy of the hand cleansing process as this will reduce the amount of 'handwashing' activity required to prevent an outbreak from occurring.

**Figure 3:**
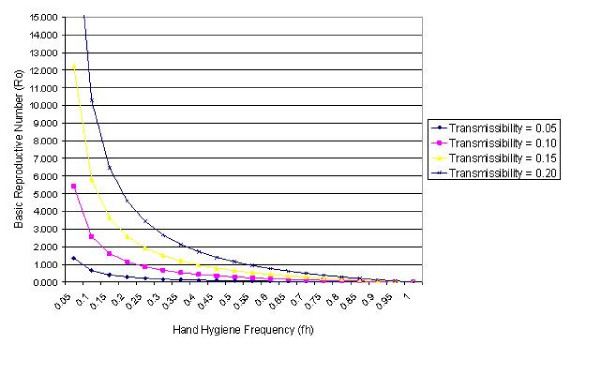
The *R*_0_-*f*_*h *_curve for a range of transmissibility values (i.e. *p*' = *p *= 0.05, 0.10, 0.15 and 0.20) when hand cleansing efficacy is 83%.

**Figure 4:**
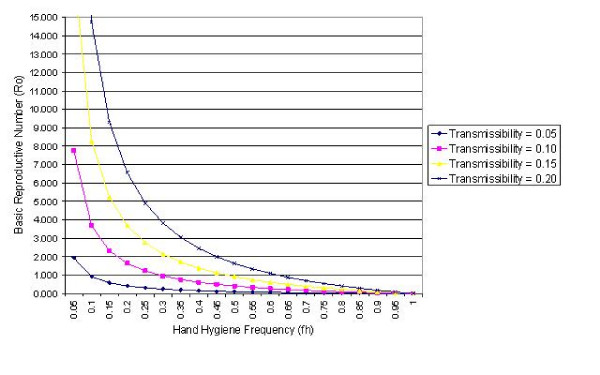
The *R*_0_-*f*_*h *_curve for a range of transmissibility values (i.e. *p*' = *p *= 0.05, 0.10, 0.15 and 0.20) when hand cleansing efficacy is 58%.

Figures 3 and 4 present *R*_0 _curves assuming various transmissibility levels in the study ward for hand cleansing efficacies of 83% and 58% respectively, and shows that the level of transmissibility greatly influences the ability of hand hygiene measures to prevent the spread of infection. At the highest transmissibility level (i.e. *p*' = *p *= 0.2) in order to ensure *R*_0 _< 1, when the efficacy is 83%, the hand cleansing frequency must be > 53.4%, whereas when *p*' = *p *= 0.1 then transmission within the ward should be avoided by compliance > 22.3%. By comparison, when the hand cleansing efficacy is only 58% and the transmissibility level is 0.2, then the compliance level required to ensure *R*_0 _< 1 is > 62.1%, while that for *p*' = *p *= 0.1 is > 29.1%. This suggests that traditional soap and water may not be sufficient to prevent outbreaks of infection when transmissibility levels are high.

**Figure 5:**
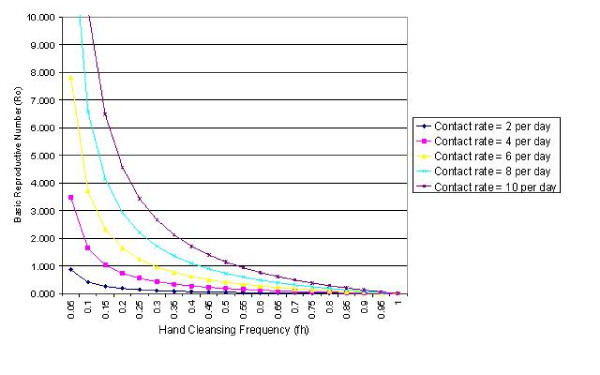
The *R*_0_-*f*_*h *_curve for a range of contact rates (i.e. *c *= 2, 4, 6, 8 and 10) when hand cleansing efficacy is 83% and transmissibility is 0.1.

The relationship between per capita daily HCW-patient contact rate and *R*_0 _is presented in Figure 5, which shows the results of analysis assuming a hand cleansing efficacy of 83%. From these data it can be seen that the contact rate has a profound influence on the transmission of infection. For moderate contact rates (i.e. up to 6 contacts per patient per HCW per day), it appears possible to prevent outbreaks occurring by ensuring that compliance is > 29.2%. However, if the contact rate increases to 10 contacts per patient per HCW per day, then is will be necessary to maintain the hand cleansing frequency at > 53.4%.

## Discussion and conclusion

While theoretical studies such as the one described in this paper can only ever be approximations of what happens in clinical practice, they can, nevertheless, yield important insights into the factors that influence the transmission of infection in hospitals. In particular, they can be useful when assessing the relative impact of various infection control measures. Provided that the data used, to a large extent, mirror the situation in a the clinical environment,, it is possible to identify general trends in the transmission dynamics. With respect to this, while the analysis presented above confirms the long held opinion that hand hygiene is an effective control measure; it also shows that the law of diminishing returns applies and that the greatest benefits are derived from the first 20% or so of compliance. Indeed, the shape of the prevalence curves presented in Figure 1 (which have the same form as those produced by Cooper *et al *[4]) suggests that little benefit is accrued from very high levels of hand cleansing. Above a certain threshold, which will vary depending on input data, the benefit of increased hand hygiene compliance appears to be minimal. In the case of the study reported here, the data show that, even under conditions of very high transmissibility, if an alcohol solution is used, it should be possible to ensure *R*_0 _< 1 when compliance is in the region 55%. This appears to confirm the findings of other researchers. For example, Cooper *et al *[4] found that under conditions of relatively high transmissibility (i.e. *p*' = *p *= 0.13) it was possible to ensure *R*_0 _< 1 with a hand cleansing frequency < 30%. McBryde *et al *[5], using a stochastic transmission model of an ICU, found that 48% hand hygiene compliance was required to ensure *R*_0 _< 1. Investigating the transmission of vancomycin-resistant enterococci on an ICU, Austin *et al *[6] found that a hand cleansing frequency of 50.5% achieved an effective reproductive number, *R*_*e *_= 0.69, well below unity (*R*_0 _= 1 when *f*_*h *_= 27.9% – extrapolated from the data of Austin). Although, these researchers assumed in their respective models a hand hygiene efficacy of 100%, their results are similar to ours, suggesting that, despite the fact that, in practise, hand cleansing is an imperfect process, it should be possible to prevent many staphylococcal outbreaks from occurring without the need to achieve excessively high hand hygiene compliance. Having said this, it is important to remember that all these researchers (including ourselves) assume the transmission of pathogens to occur exclusively via the hands of HCWs, which is unlikely to be the case [1]. Pathogens can remain viable on inanimate surfaces for long periods of time [21] and if environmental contamination in any way contributes significantly to the transmission of staphylococcal infection, then the hand hygiene compliance levels stated above may not be adequate.

The results of our study indicate that the level of hand hygiene required to ensure *R*_0 _< 1 is greatly influenced by the rate at which HCWs and patients make contact with each other, and the transmissibility of the contacts made – as these values increase, so the hand cleansing frequency required to prevent an outbreak also increases. Again, this confirms the findings of Cooper *et al *[4] who found transmissibility to be the single most influential variable in their study. In our study we assumed, as they did that, for each contact event, the probability of a HCW colonizing a patient is the same as the probability that the patient will contaminate the hands of a HCW. We did this to facilitate direct comparison with the work of Cooper *et al *[4]. This however, may not be the case, since contamination of HCWs hands is relatively transient (lasting only for a few hours), whereas patients tend to remain colonized for the length of their stay in hospital. Therefore, each colonized patient will contaminate many HCWs (*R*_*p *_>> 1), whereas a contaminated HCW will colonize patients only infrequently (*R*_*h *_<< 1) [6]. Accordingly, Austin *et al *[6] used values of *p*' = 0.40 and *p *= 0.06, which equates to a combined (*p*' × *p*) value of 0.024. Similarly, Grundmann *et al *[8] using the same methodology, adopted values of *p*' = 0.152 and *p *= 0.01, equating to a combined (*p*' × *p*) value of 0.015. By comparison, we and Cooper *et al *[4] used a combined (*p*' × *p*) value of 0.010, which, although lower than that used by the other researchers, is still of the same order of magnitude. The default HCW-patient contact rate used in our model was that suggested by Cooper *et al *[4] (i.e. 5 contacts per patient per HCW per day); considerably greater than the value of 1.38 contacts per patient per HCW per day reported by Austin *et al *[6] and somewhat less than the value of 7.6 contacts per patient per HCW per day used by Grundmann *et al *[8]. Collectively, this gives us confidence that our analysis is valid and that the variables used are realistic.

From the foregoing it can be seen that our results are consistent with earlier studies. It can therefore be concluded that it should be possible to prevent many outbreaks of staphylococcal infection through hand hygiene measures alone, even if high compliance rates are not achieved. In the study reported here it appears that compliance rates of 40% or so, should be adequate to prevent most outbreaks occurring. If this is indeed the case, then this raises questions as to why so many outbreaks of staphylococcal infection continue to occur, despite the fact that recorded hand hygiene compliance rates are generally in the region 40% [11-13]. While the reasons for this are unclear, there appear to be four possible explanations, details of which are as outlined below:

### Hawthorne Effect

While recorded hand hygiene compliance is typically in the region 40% [11-13], it may be that the Hawthorne effect is at work and that observed hand hygiene does not reflect what actually happens in reality – the implication being that general hand washing rates might be considerably lower than 40%.

### Ward Management

Another reason might be the way in which wards are organized and managed. For example, if a ward is overcrowded or under-staffed, then those nurses on duty will have to attend to more patients than usual and so the HCW-patient contact rate is likely to rise and with it the hand cleansing frequency required to ensure *R*_0 _< 1. This was graphically illustrated by Grundmann *et al *[8], who, in their study on an ICU, found exposure to relative staff deficit to be the only factor significantly associated with MRSA transmission. Indeed, they predicted that it would require an additional 12% improvement in adherence to hand hygiene policies to compensate for staff shortages. Given that during this study, observed hand hygiene compliance was on average 59%, the investigators concluded that under conditions of overcrowding and high workload, it would be impossible for the nursing staff to achieve the required additional compliance. The HCW-patient contact rate is also influenced by the way in which nurses are organized. Beggs *et al *[7] demonstrated that if nursing staff are allowed to mix freely with patients, then the number of potential transmission routes will be high, leading to increased need for hand cleansing. In order to minimise the number of transmission routes it is necessary to cohort the nursing staff so that they cannot transfer pathogens between different groups of patients. Other studies have reached similar conclusions [5,6,12,20,22] – the higher the level of cohorting, the fewer the number of contacts between patients.

### Colonized Admissions

**Figure 6:**
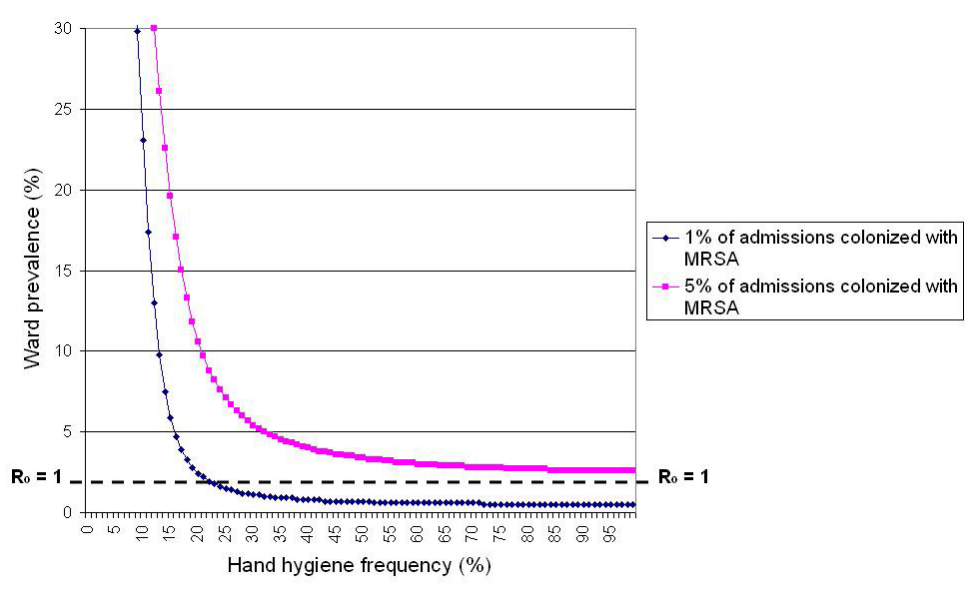
The impact of variations in the proportion of admissions already colonized with MRSA on the prevalence of infection, assuming λ' = 83%.

Figure 5 shows the effect of variations in the proportion of admissions already colonized with MRSA on the prevalence of infection, assuming an average hand cleansing efficacy, λ', of 83%. From this it can be seen that as the number of colonized patients entering the ward increases, so the model predicts that it is not possible to completely eradicate infection through hand hygiene measures alone. No matter the level of hand hygiene compliance, there will always be a residual level of infection which is difficult to eradicate. Therefore, if the number of colonized patients admitted to hospitals is high, then this might explain why increased hand hygiene compliance is failing to control the spread of staphylococcal infection. From Figure 6 it can be seen that when the proportion of MRSA colonized patients entering hospital is 5%, the model predicts that *R*_*o *_> 1, no matter the level of hand hygiene compliance.

### Environmental Contamination

Another reason why improved hand hygiene compliance might not deliver the hoped-for results might be because environmental contamination may be important in the transmission of staphylococcal infection. A number of researchers have demonstrated that widespread environmental contamination can occur as a result of MRSA infection/colonization [22-26]. For example, Boyce *et al*. [23] in a study in a US hospital, found environmental contamination in 73% of the rooms of MRSA infected patients and 69% of colonized patients. Indeed, they found 27% of the surfaces sampled in rooms containing MRSA-infected patients to be contaminated with MRSA, with frequently contaminated objects including the floor, bed linen, patients' gowns, over-bed tables and sphygmomanometer cuffs. Others have cultured MRSA from the air in patient rooms [22,24,25], and Wilson *et al *[26] observed a strong correlation between the presence of MRSA patients and air samples yielding MRSA in an ICU. Although many accounts of environmental contamination have been published it has proved very difficult to determine causality, and it is not known to what extent environmental contamination contributes towards the transmission of staphylococcal infection. However, it is thought that such contamination may seed environmental reservoirs resulting in increased sporadic infection [23]. If this is indeed the case, then it might explain why staphylococcal infection has been so difficult to eradicate using hand hygiene measures alone. Accordingly we would recommend that environmental contamination with the bacterium is considered in future models of *S. aureus *transmission within the hospital setting.

In this paper we used a deterministic model to analyse the transmission dynamics of staphylococcal infection. While this approach has validity, it is not without drawbacks. Deterministic dynamic models predict that the persistence of infection is only possible above a certain critical threshold, *R*_*o *_= 1 (i.e. one infected or colonized patient must transmit the pathogen to, on average, at least one other patient). However in reality, outbreaks can occur even when these threshold conditions appear not to be met (i.e. when *R*_*o *_< 1). Conversely, outbreaks may die out despite *R*_*o *_> 1. This is because stochastic effects often dominate in small populations, such as those found within hospitals [27]. This means that while most outbreaks should be controlled when *R*_*o *_< 1, some will not. In a few cases, chance events will be such that outbreaks of staphylococcal infection may occur despite the presence of stringent control measures. While the use of stochastic modelling would have yielded data on the variance of the transmission dynamics, the focus of our paper is on the impact of hand hygiene on average prevalence curves, which can be predicted using deterministic methods – hence the strategy adopted in this paper.

## Competing interests

The authors declare that they have no competing interests.

## Authors' contributions

CBB and SJS designed the study. SJS constructed the computer model and KGK advised on the clinical aspects of the study. CBB wrote the manuscript with major contributions from other authors. All authors have read and approved the final manuscript.

## Appendix

In the model *x *represents the number of susceptible patients and *y *the number of colonized patients, with the total number of patients in the ward being a constant *n *(i.e. *x *+ *y *= *n*). Similarly, the total number of carers is *n*', with *x*' representing those HCWs not carrying the pathogen and *y*' representing those who are temporarily colonized, where *x*' + *y*' = *n*'. The removal rate for uncolonized patients is μ, which includes removals due to death, transfers to other wards, and discharges. The detection rate of colonized patients is γ, which is assumed to be the rate at which colonized patients are deliberately removed from the ward. With regard to the cleansing of hands, the handwashing rate is μ' and the average efficacy of each handwashing event is λ'. The rate at which contamination is removed from the hands of HCWs on the ward is therefore μ' *y*'λ'.

In the model it is assumed that each patient requires a contact from a HCW in a given time interval with a given probability and that no superfluous contacts are made. The mean number of contacts required by each patient per day is defined as *c*, with *p *being the probability that a patient becomes colonized on contact with a contaminated HCW, and *p*' to be the probability that a HCW becomes contaminated on contact with a colonized patient. In the model we let β = *cp *and β' = *cp*'. The rates at which contacts occur that can potentially result in colonization are therefore β*x *(for patient colonization) and β' *y *(for HCW contamination). Since a fraction *y*'/*n*' of contacts will be with colonized HCWs, and *x*'/*n*' with susceptible HCWs, the rates for patient and carer colonization will be β*xy*'/*n*' and β'*x*'*y*/*n*' respectively. The proportion of patients admitted to the ward who are colonized or infected is σ, where(0 ≤ σ ≤ 1).

Based on the above, the model solves the following differential equations to determine the spread of infection/colonization on the ward.

In the model the rate of change of colonized patients and contaminated HCWs is given by:

(1a) $$\frac{dy}{dt}=\sigma \left(\mu x+\mu y+\gamma y\right)+\beta x\frac{y^{\prime }}{n^{\prime }}-y(\mu +\gamma )$$

(2a) $$\frac{dy^{\prime }}{dt}=\beta^{\prime }y\frac{x^{\prime }}{n^{\prime }}-\mu^{\prime }\lambda^{\prime }y^{\prime }$$

For this system of equations, the basic reproductive number, *R*_0_, is defined as:

(3a) $$R_{0}=\frac{(n-1)\beta \beta^{\prime }}{(\mu +\gamma )n^{\prime }\mu^{\prime }\lambda^{\prime }}$$

## Pre-publication history

The pre-publication history for this paper can be accessed here:


